# Integration of pharmacophore-based virtual screening, molecular docking, ADMET analysis, and MD simulation for targeting EGFR: A comprehensive drug discovery study using commercial databases

**DOI:** 10.1371/journal.pone.0311527

**Published:** 2024-12-09

**Authors:** Abdullah R. Alanzi, Ashaimaa Y. Moussa, Mohammed S. Alsalhi, Tayyab Nawaz, Ijaz Ali

**Affiliations:** 1 Department of Pharmacognosy, College of Pharmacy, King Saud University, Riyadh, Saudi Arabia; 2 Faculty of Pharmacy, Department of Pharmacognosy, Ain-Shams University, Cairo, Egypt; 3 Department of Pharmaceutical Chemistry, College of Pharmacy, King Saud University, Riyadh, Saudi Arabia; 4 Department of Mathematics, University of Illinois at Urbana Champaign, Champaign, IL, United States of America; 5 Centre for Applied Mathematics and Bioinformatics (CAMB), Gulf University for Science and Technology, Hawally, Kuwait; University of Mashreq, IRAQ

## Abstract

The epidermal growth factor receptor (EGFR), a crucial component of cellular signaling pathways, is frequently dysregulated in a range of cancers. EGFR targeting has become a viable approach in the development of anti-cancer medications. This study employs an integrated approach to drug discovery, combining multiple computational methodologies to identify potential EGFR inhibitors. The co-crystal ligand for the EGFR protein (R85) (PDB ID: 7AEI) was employed as a model for developing pharmacophore hypotheses. Nine databases underwent a ligand-based virtual screening, and 1271 hits meeting the screening criteria were chosen. EGFR protein crystal structure was obtained from the PDB database (PDB ID: 7AEI) and prepared. The hit compounds identified during virtual screening were docked to the prepared EGFR receptor to predict binding affinities by using the glide tool’s standard precision mode. The top ten compounds were chosen, and their affinities of binding ranged from -7.691 to -7.338 kcal/mol. The ADMET properties of the selected compounds were predicted, and three compounds MCULE-6473175764, CSC048452634, and CSC070083626 showed better QPPCaco values compared to other identified compounds, so these were selected for further stability analysis. To confirm the stability of the protein-ligand complexes, a 200 ns molecular dynamics (MD) simulation was run using the binding sites of the top three compounds against the EGFR receptor. These results suggest that the selected compounds may be lead compounds in suppressing the biological activity of EGFR, additional experimental investigation is required.

## Introduction

The transmembrane glycoprotein known as epidermal growth factor receptor (EGFR) has an intracellular tyrosine kinase domain in addition to an external EGF binding region. It governs cellular proliferation and signaling pathways [1]. It has been found that EGFR is overexpressed in a variety of cancer cells, including those from the head and neck, breast, esophagus, and lung. EGFR is a prospective target for anti-cancer treatment because it plays a crucial role in the incidence and development of cancer [2]. The origin and growth of tumor cells are significantly influenced by abnormal EGFR activity, which results in cellular responses such cell death and proliferation. EFGR activation and overexpression are associated with worse patient outcomes in cancer. EGFR is a major target for therapy in clinical oncology [3].

Numerous EGFR inhibitors have been identified and given clinical approval in recent years. Two primary types of EGFR inhibitors are currently being studied: small molecule tyrosine kinase inhibitors (TKIs) and monoclonal antibodies (mAb) [4]. Now, several small-molecule TKIs that target EGFR have been developed [5,6]. As first-generation representative EGFR-TKIs, erlotinib (IC50 = 80 nM) and gefitinib (IC50 = 23–79 nM) were clinically studied in contrast to conventional chemotherapy [7]. These medications work as competitive ATP inhibitors that are reversible to stop EGFR autophosphorylation [8]. The second-generation EGFR-TKI afatinib (IC50 = 0.5 nM) has gained immense popularity in the treatment of breast cancer [9]. Additionally, third-generation TKI Osimertinib (IC50 = 12 nM) targets resistance mutations in EGFR-T790M that arise from the usage of first-generation TKIs [10,11].

Research on EGFR natural product inhibitors has suggested that chalcone, sesquiterpene lactones, and phenolic compounds together may improve the efficacy of small molecule inhibitors and increase drug sensitivity [12]. Abdel Gawad and colleagues synthesized novel phenolic compounds that may function as COX-2 and EGFR inhibitors. After conducting an examination, they discovered compounds C4 and G4, which had IC50 values of 0.9 and 0.5 μM, respectively, and shown significant inhibitory action [13]. Abou-Zied HA et al. developed a new xanthine derivative including the chalcone component to investigate potential EGFR inhibitors. Compound 11, which had an IC50 value of 0.3 μM against the target enzyme, was successfully obtained [14]. Furthermore, a remarkable research by Nerdy et al. revealed that by suppressing the expression of EGFR, sesquiterpene lactones from veronica amygdaline extract demonstrated potential anticarcinogenic properties [15].

**Fig 1:**
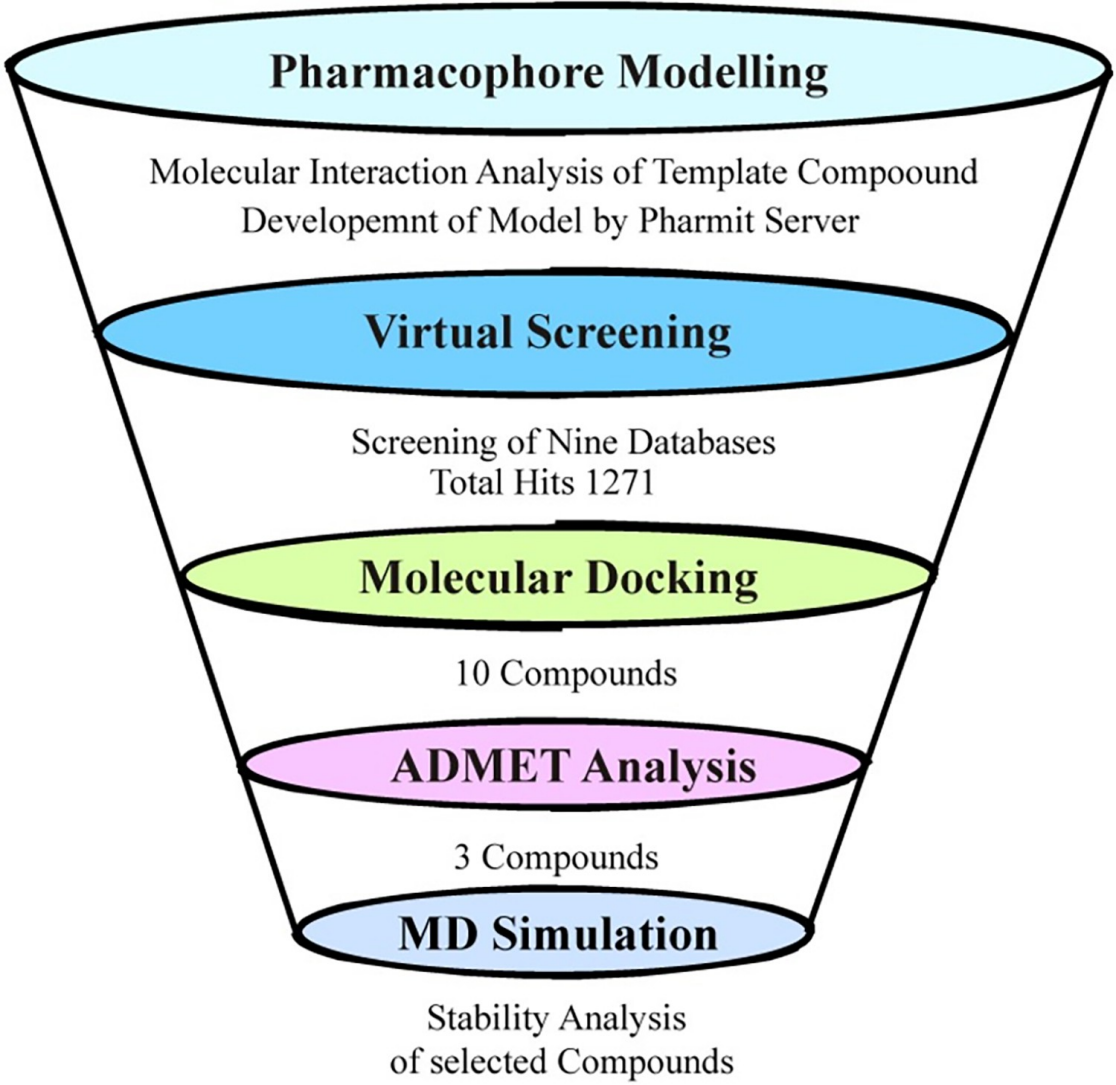
The workflow of the study.

Even with their strong action, EGFR inhibitors have drawbacks include poor in vivo and cellular effectiveness as well as drug resistance. Thus, the creation of new EGFR inhibitors requires urgent attention [16]. The combination of computational techniques, including pharmacophore-based virtual screening, molecular docking, ADMET analysis, and MD simulation, provides a comprehensive strategy for discovering novel EGFR inhibitors with improved efficacy and fewer side effects. Hence, this study was designed to identify novel EGFR inhibitors utilizing these computational techniques. The multifaceted methodology of the current study makes it stand apart. When compared to traditional in silico research, it provides a more complete and reliable drug discovery pipeline by merging molecular docking, enhanced pharmacophore modeling, detailed ADMET profiling, and molecular dynamics simulations. Furthermore, the utilization of comprehensive commercial databases provides access to a broader spectrum of chemical entities, increasing the possibility for discovering novel and powerful EGFR inhibitors. This comprehensive methodology differs from previous investigations, which usually focused on a smaller range of computational tools and less diversified chemical libraries. The workflow of the study is shown in Fig 1.

## Materials & methods

### Pharmacophore Modelling

A pharmacophore model can be described as a chemical template that comprises the essential structural features of biologically active compounds. The structural features of an active compound are utilized to generate pharmacophore model which then processed to conduct the screening of large chemical databases [17]. We developed a ligand-based pharmacophore model using the chemical features of a co-crystal ligand (R85) of Epidermal growth factor receptor (PDB ID: 7AEI) by using the Pharmit server [18,19]. The server offers a protocol to screen the chemical databases based on the developed pharmacophoric features. The chemical structure of R85 ligand was used to develop the pharmacophore model based on its interactions with EGFR binding pocket.

### Virtual screening

The model used for virtual screening was created using the four pharmacophoric features of the co-crystal ligand: hydrophobic, aromatic, hydrogen bond acceptor, and donor of hydrogen bonds. The parameters of the virtual screening were set based on the Lipinski’s rule [20]: molecular weight < 500, hydrogen bond donor (HBD) < 5, hydrogen bond acceptor (HBA) < 10, and LogP < 5. For the virtual screening, the following databases were explored: ZINC, Lab Network, PubChem, Moleport, Enamine, MCULE, Chemspace, ChemDiv, and CHEMBL.

### Ligand preparation

A total of 1271 hits obtained from the pharmacophore based virtual screening were prepared by using the LigPrep program from Schrödinger’s Maestro [21]. For every ligand, conformers were generated, and geometries were optimized using LigPrep. The OPLS_2005 forcefield was utilized to modify the ligands’ geometry to guarantee that they were in conformations that were energetically favorable [22]. By reducing the energy of the compounds, any unfavorable interactions or strained geometry were removed.

### Molecular docking

The molecular docking of the prepared hits was conducted against the EGFR receptor. The crystal structure of EGFR protein was retrieved from PDB database (PDB ID: 7AEI) and prepared for the docking using Protein Preparation Wizard [23]. There were several processes involved in the preparation of protein. Bond orders were set, disulfide bonds were created, and zero-order metal bonds were allocated. Additionally, hydrogen was added to the protein structures. All additional water molecules and ligands were eliminated from the crystal structures. Using the PROPKA program, We calculated the protein ionizable groups’ pKa values [24], and proteins’ hydrogen bond networks were optimized at pH 7.0. Lastly, the OPLS_2005 forcefield was used to reduce the energy of the protein structure. After the protein was prepared, a site-specific docking 3D grid was built at X, Y, Z coordinates of 8.32, 6.48, and 9.1. With the Glide docking module in SP (Standard Precision) mode, the prepared ligands were docked at particular regions on the prepared protein structure [25]. After examination, the docked ligands were selected according to their glide scores.

### ADMET analysis

To determine their ADMET (absorption, distribution, metabolism, excretion, and toxicity) and physicochemical properties, the docked ligands underwent a comprehensive analysis. To achieve this, Maestro’s QikProp tool was employed, providing predictions for various attributes based on the ligands’ molecular structures [26]. Molecular weight, hydrogen bond acceptors, Hydrogen bond donors, QPlogBB, QPPCaco, QPlogKhsa, QPlogPo/w, and QPlogHERG were important characteristics. Hydrogen bond donors and acceptors are metrics that quantify the amount of atom centers and hydrogen atoms available for participating in interactions involving hydrogen bonds. The logarithm of the octanol and water partition coefficient is predicted by QPlogPo/w, which provides information about the compound’s membrane permeability and hydrophobicity. QPlogHERG assesses the potential of a ligand to block the hERG potassium channel, providing information about the likelihood of cardiac toxicity. QPPCaco is a model for intestinal absorption that determines a compound’s permeability over the monolayer of Caco-2 cells. The substance’s ability to penetrate the blood-brain barrier and reach the central nervous system is indicated by QPlogBB, which forecasts the BBB partition coefficient’s logarithm. Finally, the logarithm of the binding affinity to human serum albumin, a necessary protein that influences drug distribution and binding efficiency, is determined by QPlogKhsa.

### MD simulation

Desmond was used to perform MD simulations of selected compounds for 200 ns [27]. We performed Molecular Dynamics simulations to evaluate the stability of the protein and ligand complexes. Molecular Dynamics simulation was used to evaluate the stability of complexes after several stages, including preprocessing, optimization, and reduction. Minimization was done using the OPLS_2005 force field [22]. The compounds were solvated in a periodic box with a 10 Å size containing the TIP3P water molecules [28]. Neutralization of the systems was done by adding counter ions and 0.15 M NaCl salt as needed to mimic physiological conditions. A pressure of 1 atm and a temperature of 300 K were set using the NPT ensemble. The systems went through a relaxing period before the simulation started. Trajectories were recorded and saved at 40 ps intervals during the simulation, allowing for a later study of the outcomes.

## Results

### Pharmacophore modelling and virtual screening

**Fig 2:**
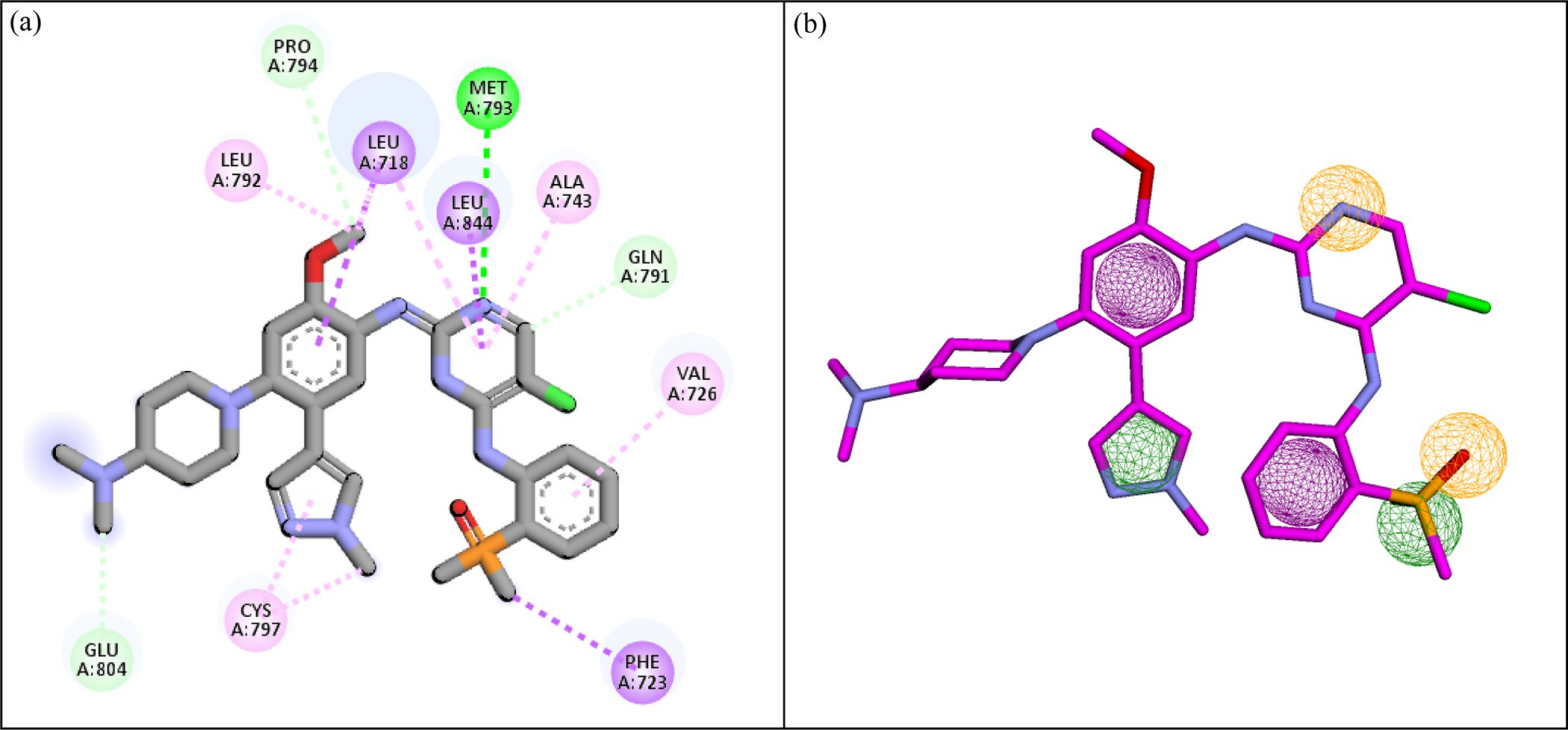
a) The molecular interactions of R85 with EGFR used for developing pharmacophore model. (b)The pharmacophore query model generated by Pharmit server. Green spheres show hydrophobic group, purple shows the aromatic rings, gray shows the hydrogen bond donor while orange sphere shows the hydrogen bond acceptor.

The pharmacophoric features of R85 ligand involved in the molecular interactions with EGFR protein were used to develop the pharmacophore query model (Fig 2A). There was a total of six features which were used to generate the query model (Fig 2B). The X, Y, and Z coordinates of the features are shown in Table 1. Based on these features, ligand based virtual screening of nine databases was conducted and the hits meeting the screening criteria were selected (Table 2). There was a total of 1271 hits collectively obtained from the nine databases. Among these hits, MCULE database produced the highest number of hits.

**Table 1:** The pharmacophoric features their coordinates, generated by Pharmit server.

Pharmacophoric Features	X	Y	Z	Radius
Hydrophobic	-3.66	50.57	-24.24	1
Hydrophobic	-2.67	49.53	-17.55	1
Hydrogen Acceptor	-2.03	51.60	-24.87	1
Aromatic	0.79	51.54	-16.81	1
Aromatic	-1.49	49.07	-21.78	1
Hydrogen Acceptor	2.12	54.29	-20.85	1

**Table 2:** The generated hits from each database based on ligand-based virtual screening.

Sr.	Databases	Hits
1	CHEMBL	26
2	ChemDiv	12
3	Chemspace	157
4	Enamine	35
5	MCULE	446
6	MolPort	42
7	PubChem	427
8	LabNetwork	9
9	ZINC	117
	Total	1271

### Molecular docking

**Fig 3:**
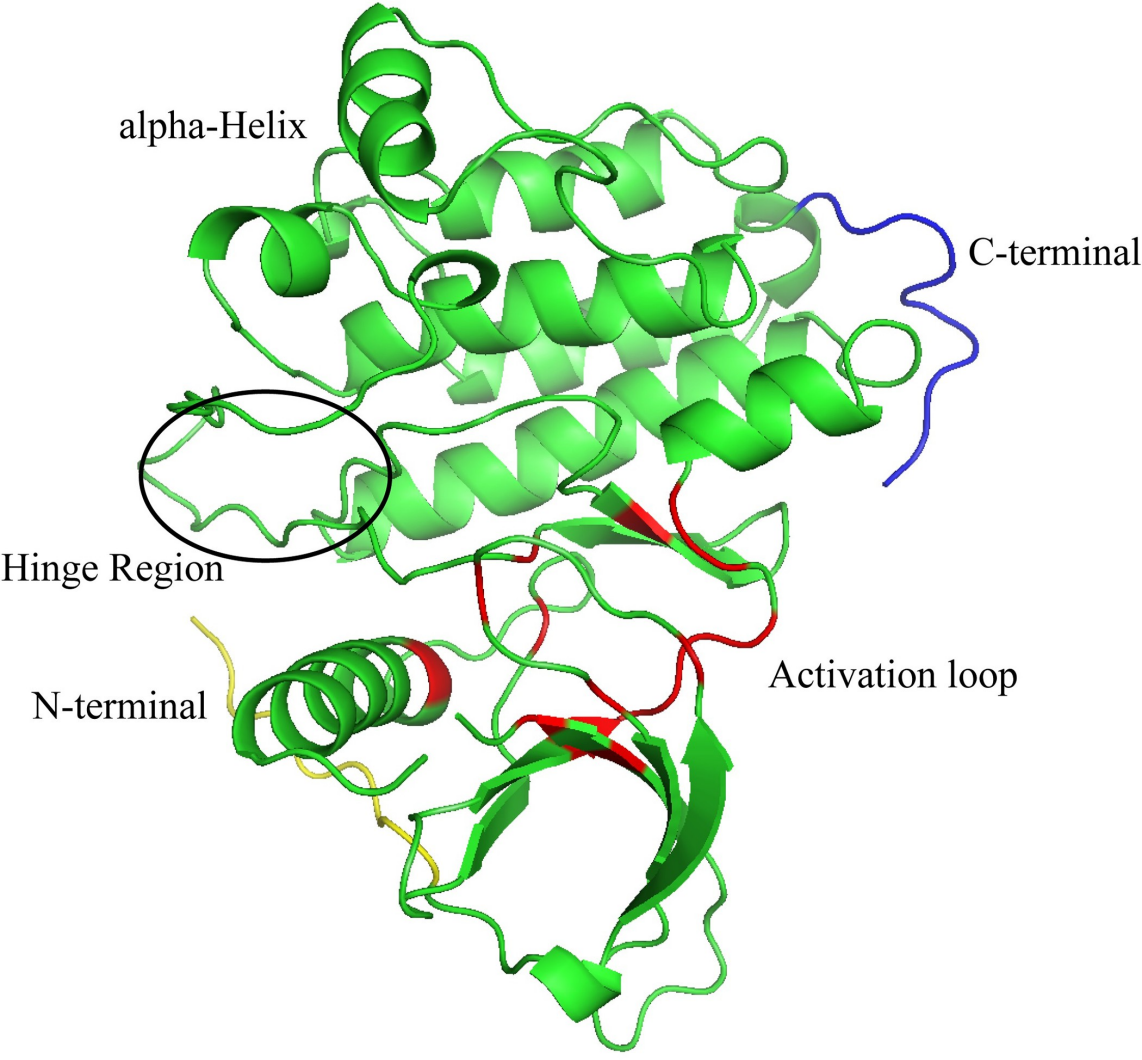
The representation of protein topology. The binding pocket is shown in red color, hinge region is highlighted with circle, N- and C- terminals are shown with yellow and blue colors, respectively.

The hit compounds generated during virtual screening were docked to the prepared EGFR receptor to predict the binding affinities by using the standard precision mode of glide tool. The EGFR kinase has an activation loop near the binding site (highlighted with the red color in Fig 3). Moreover, the hinge region plays a significant role in the hydrogen bonding and stabilization of the complex. The topology of the protein plays a key role in the molecular interactions of the compounds with protein resulting in the good binding affinity of compounds. Based on the binding affinities, the top ten compounds were selected for further analysis (Table 3). The binding affinities of the selected compounds were in the range of -7.691 to -7.338 kcal/mol. Further, the co-crystal ligand R85 and known ATP-competitive EGFR inhibitors were docked with the protein and the binding affinities were compared with the hits. The binding affinities of the inhibitors and R85 were in the range of -7.18 to -5.60 kcal/mol. The binding affinities of the selected compounds indicated that these have the probability of inhibiting the function of EGFR protein.

**Table 3:** The binding affinities of the selected compounds along with their structures.

Sr.	Compound code	Glide score (kcal/mol)
1	MCULE-6473175764	-7.69
2	PubChem-70897620	-7.65
3	CSC081909901	-7.60
4	MCULE-2074984553	-7.57
5	CHEMBL2440371	-7.44
6	PubChem-90330948	-7.42
7	CSC048452634	-7.37
8	MCULE-5325020620	-7.36
9	PubChem-123467855	-7.36
10	CSC070083626	-7.33
11	R85	-7.18
12	Gefitinib	-6.25
13	Erlotinib	-5.60
14	Afatinib	-6.41
15	Osimertinib	-6.94

### Molecular interactions analysis

**Fig 4:**
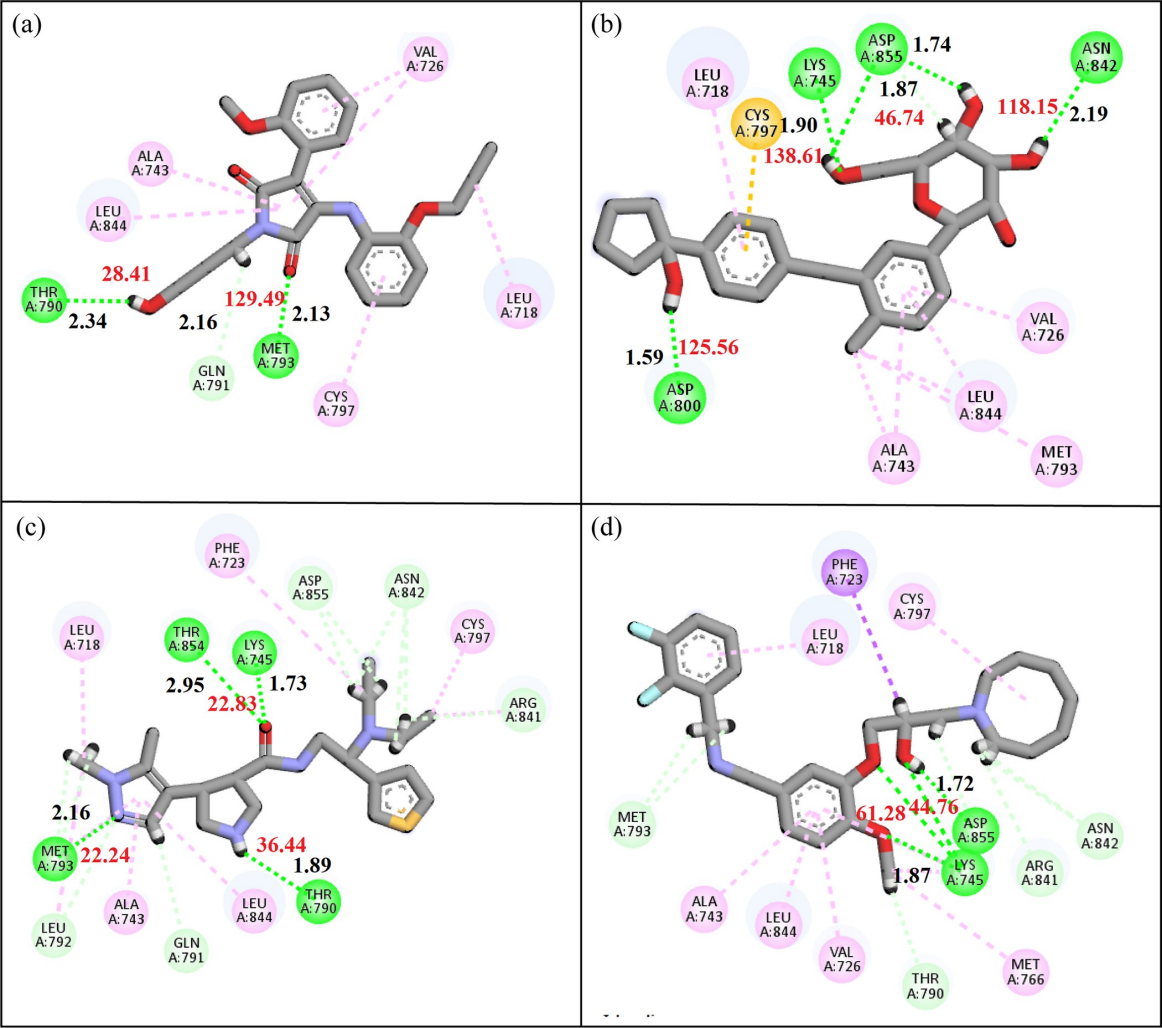
The molecular interactions of the selected compounds. (a) MCULE-6473175764, (b) PubChem-70897620, (c) CSC081909901, (d) MCULE-2074984553. Green spheres show the conventional hydrogen bonds, gray shows the carbon hydrogen bonds, orange shows the Pi-Cation interactions, Cyan shows the halogen, and magenta shows the alkyl interactions. The black labeled numbers show hydrogen bond distances while red numbers show the bond angles.

The molecular interactions of the selected hits with the binding pocket of EGFR receptor were analyzed using the Discover Studio client tool. Conventional hydrogen bonds, carbon hydrogen bonds, van der Waal interactions, Pi-Sulfur, Amide Pi-Stacked, Halogen, and Alkyl interactions were the primary interactions that were detected. These interactions play a pivotal role in determining the binding affinities and docking scores for each of the top candidate compounds. Notably, the formation of intermolecular hydrogen bonds between the ligand and the amino acid within the active sites has a significant impact on the overall strength of the resulting complex. The distance between the hydrogen bond forming atoms and bond angles play a significant role in the strength of hydrogen bonds, so these were also measured. Consequently, these interactions consistently enhance the docking results [29]. **MCULE-6473175764** formed two conventional hydrogen bonds with Thr790, Met793, one carbon hydrogen bond with Gln791, and five alkyl interactions with Leu844, Ala743, Val726, Leu718, Cys797 (Fig 4A). **PubChem-70897620** formed four conventional hydrogen bonds with Asp800, Lys745, Asp855, Asn842, one Pi-Sulfur interaction with Cys797, and five alkyl interactions with Leu718, Ala743, Leu844, Met793, Val726 (Fig 4B). Similarly, **MCULE-3666578374** made four conventional hydrogen bonds with Met793, Thr854, Lys745, Thr790, five carbon hydrogen bonds with Leu792, Gln791, Asp855, Asn842, Arg841, and five alkyl interactions with Leu718, Phe723, Cys797, Leu844, Ala743 (Fig 4C). Lastly, **MCULE-2074984553** made two conventional hydrogen bonds with Lys745, Asp855, four carbon hydrogen bonds with Met793, Arg841, Asn842, Thr790, one Pi-Sigma interaction with Phe723, and six alkyl interactions with Ala723, Leu844, Val726, Met766, Cys797, Leu718 (Fig 4D). On comparison with the hits, the co-crystal structure made four Hydrogen Bonds with Met793, Pro794, Gln791, Glu804, three Pi-Sigma interactions with Leu718, Leu844, Phe723, and four Alkyl interactions with Leu792, Ala743, Val726, Cys797. The molecular interactions of the several ATP-competitive EGFR inhibitors were also analyzed with the protein and compared with the hits. The molecular interactions known inhibitors and other hits are shown in Table 4.

**Table 4:** The molecular interactions of the selected docked compounds with EGFR binding site residues.

Sr.	Compound code	Interactions
1	MCULE-6473175764	**Conventional Hydrogen Bond:** Thr790, Met793 **Carbon Hydrogen Bond:** Gln791 **Alkyl:** Leu844, Ala743, Val726, Leu718, Cys797
2	PubChem-70897620	**Conventional Hydrogen Bond:** Asp800, Lys745, Asp855, Asn842 **Pi-Sulfur:** Cys797 **Alkyl:** Leu718, Ala743, Leu844, Met793, Val726
3	CSC081909901	**Conventional Hydrogen Bond:** Met793, Thr854, Lys745, Thr790 **Carbon Hydrogen Bond:** Leu792, Gln791, Asp855, Asn842, Arg841 **Alkyl:** Leu718, Phe723, Cys797, Leu844, Ala743
4	MCULE-2074984553	**Conventional Hydrogen Bond:** Lys745, Asp855 **Carbon Hydrogen Bond:** Met793, Arg841, Asn842, Thr790 **Pi-Sigma:** Phe723 **Alkyl:** Ala723, Leu844, Val726, Met766, Cys797, Leu718
5	CHEMBL2440371	**Conventional Hydrogen Bond:** Arg841, Asn842, Asp855 **Carbon Hydrogen Bond:** Met793 **Pi-Sigma:** Leu718 **Alkyl:** Val726, Leu844, Ala743
6	PubChem-90330948	**Conventional Hydrogen Bond:** Met793, Ser720, Asp800 **Pi-Sigma:** Val726 **Alkyl:** leu844, Ala743, Cys797
7	CSC048452634	**Conventional Hydrogen Bond:** Asp800, Met793 **Carbon Hydrogen Bond:** Gly719, Leu718 **Alkyl:** Ala743, Leu792, Leu844, Val726, Cys797
8	MCULE-5325020620	**Conventional Hydrogen Bond:** Thr854 **Alkyl:** Val726, Leu718, Ala743, Leu844
9	PubChem-123467855	**Conventional Hydrogen Bond:** Asp800, Cys797, Leu718, Lys745 **Alkyl:** Cys775, Met766, Ala743, Leu844, Val726, Leu792
10	CSC070083626	**Conventional Hydrogen Bond:** Met793, Asp855, Lys745 **Carbon Hydrogen Bond**: Asp837 **Alkyl:** Leu718, Leu844, Val726, Arg841, Phe723
11	R85	**Conventional Hydrogen Bond:** Met793 **Carbon Hydrogen Bond**: Pro794, Gln791, Glu804 **Pi-Sigma:** Leu718, Leu844, Phe723 **Alkyl:** Leu792, Ala743, Val726, Cys797
12	Gefitinib	**Conventional Hydrogen Bond:** Lys745, Asn842 **Carbon Hydrogen Bond**: Gln791 **Alkyl:** Leu718, Leu792, Met793, Leu844, Met766, Val726, Met766 **Salt-Bridge:** Asp855
13	Erlotinib	**Conventional Hydrogen Bond:** Lys745, Thr854 **Carbon Hydrogen Bond**: Thr790, Asp855, Glu762 **Alkyl:** Leu718, Leu792, Val726, Cys797, Leu844
14	Afatinib	**Conventional Hydrogen Bond:** Met793, Asn842, Thr854 **Pi-Sigma:** Leu718 **Alkyl:** Leu844, Ala743, Val726, Lys745 **Salt-Bridge:** Asp855, Asp837
15	Osimertinib	**Conventional Hydrogen Bond:** Met793, Cys797 **Carbon Hydrogen Bond**: Leu718, Pro794, Gln791 **Pi-Sigma:** Val726 **Alkyl:** Phe723, Leu792, Ala743, Leu844 **Salt-Bridge:** Asp800

### ADMET analysis

QikProp was used to predict the ADMET characteristics of the selected compounds, and it was found that the expected values fell within an acceptable range. The molecular weight of a compound indicates its easy distribution in the cells so the compounds with less weight can easily distribute in the body as compared to the compounds with higher weight. In this regard, a criterion of 500 g/mol was set, and all the molecular weights of all selected compounds fall within this range. QPlogPo/w determines the octanol/water partition coefficient, a value within a range of –2.0 to 6.5 is good. The values of selected hits fall within this range. The compounds that were selected had anticipated ADMET qualities that are within the acceptable range, as demonstrated in Table 5. Three compounds **MCULE-6473175764**, **CSC048452634**, and **CSC070083626** showed better QPPCaco values compared to other identified compounds. So, these compounds were selected for further stability analysis.

**Table 5:** The ADMET properties of top ten compounds.

Compounds	MW	HBD	HBA	QPlogPo/w	QPlogHERG	QPPCaco	QPlogBB	QPlogKhsa
**MCULE-6473175764**	**410.469**	**2**	**6.7**	**4.137**	**-5.612**	**939.893**	-1.109	0.453
PubChem-70897620	428.524	5	9.25	2.29	-5.471	213.196	-1.729	0.008
CSC081909901	389.558	1	6	2.611	-4.923	52.606	0.147	0.231
MCULE-2074984553	448.552	2	6.7	4.148	-6.838	147.52	0.235	0.611
CHEMBL2440371	394.851	4	9.25	1.734	-5.126	292.16	-1.377	-0.374
PubChem-90330948	406.862	3	10.5	1.57	-5.307	222.114	-1.457	-0.356
**CSC048452634**	**388.413**	**2**	**4.45**	**3.982**	**-5.555**	**1255.923**	-0.09	0.499
MCULE-5325020620	340.381	0	9.25	0.842	-4.236	446.307	-0.666	-1.078
PubChem-123467855	433.931	5	9.5	2	-5.073	138.898	-1.809	-0.198
**CSC070083626**	**385.459**	**2**	**7.7**	**3.22**	**-5.361**	**712.29**	-1.279	0.11

"QPlogHERG" (<-5), "QPlogPo/w" (-2.0 to 6.5), "QPlogBB" (-3.0 to 1.2), "QPPCaco" (<25 poor, >500 great), and "QPlogKhsa" (-1.5 to 1.5).

### Binding pose analysis

**Fig 5:**
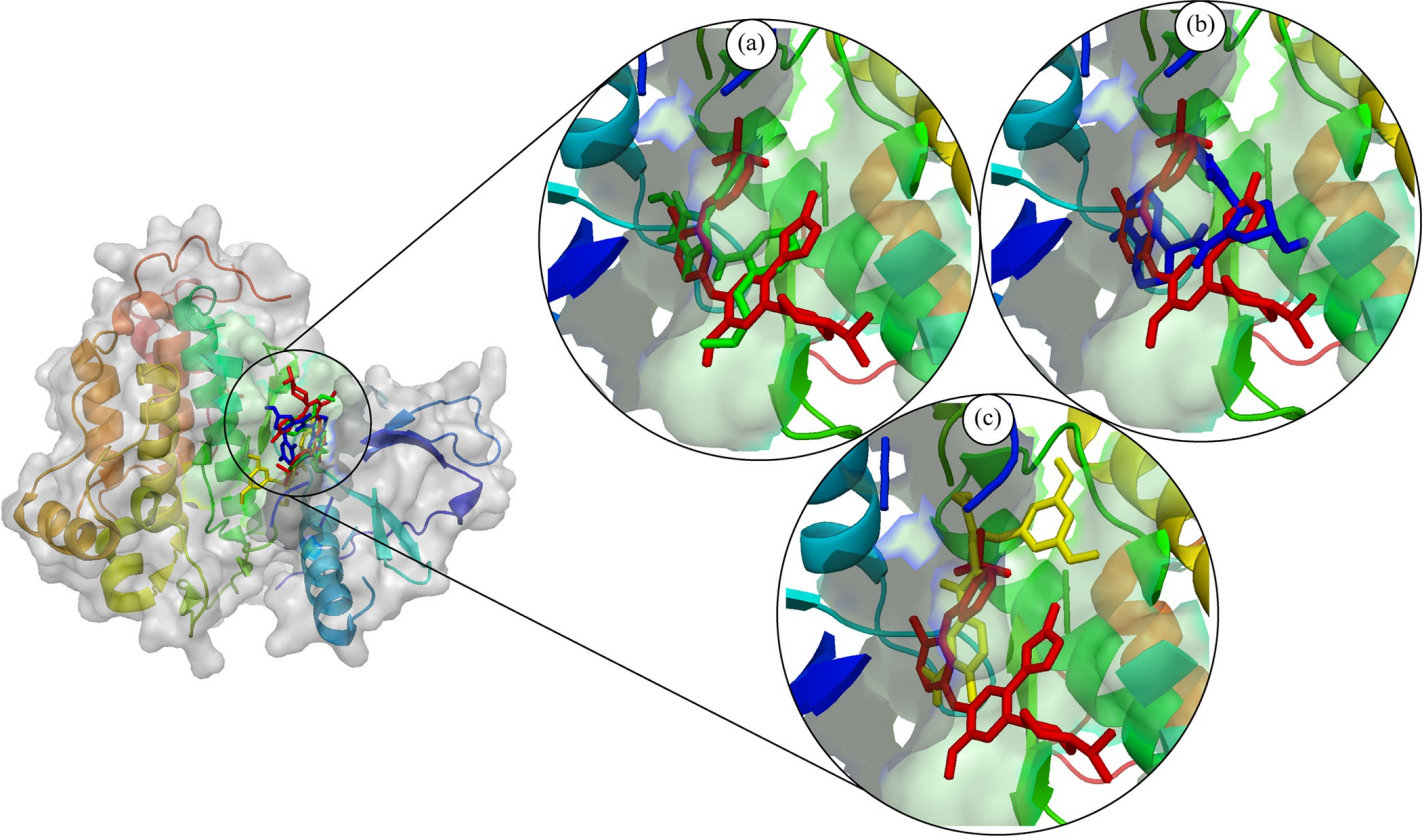
The plausible binding modes of the selected compounds aligned on the co-crystal ligand (red sticks). (a) MCULE-6473175764 (Green sticks), (b) CSC048452634 (Blue sticks), (c) CSC070083626 (Yellow sticks).

**Fig 6:**
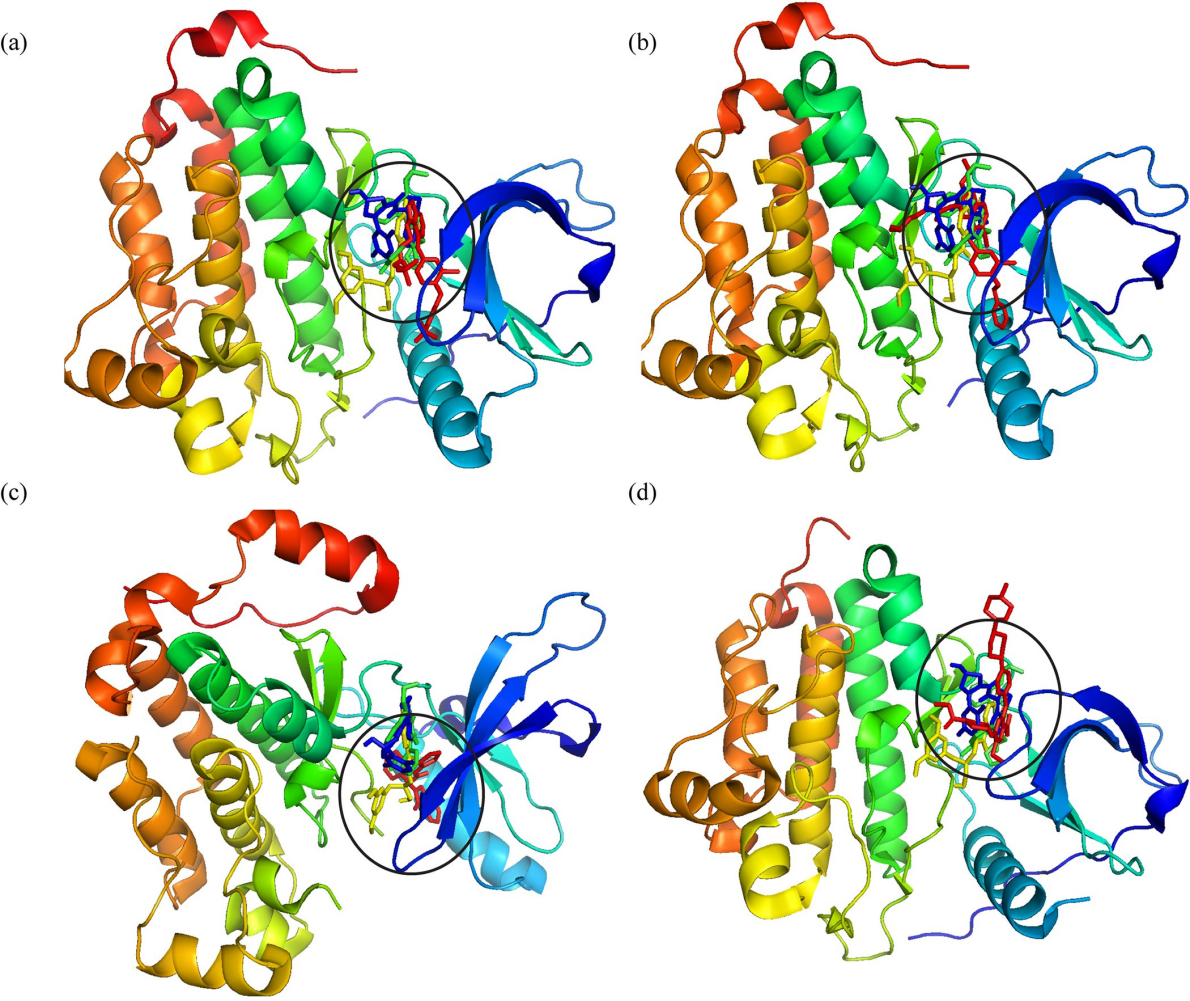
The plausible binding modes of hits in the binding pockets of mutant proteins. The co-crystal ligand of each protein is shown with red sticks, MCULE-6473175764 (Green sticks), CSC048452634 (Blue sticks), CSC070083626 (Yellow sticks). (a) 3W2O, (b) 3W2Q, (c) 5D41, and (d) 5Y9T.

After the molecular interaction analysis, the binding poses of the selected compounds were identified by aligning them on the co-crystal ligands. According to the analysis, the docked compounds had a comparable binding mechanism and were completely aligned on the co-crystal ligand (Fig 5). The plausible binding modes of the hits were further analyzed with the various mutants of EGFR protein i.e., (PDB ID: 3W2O, 3W2Q, 5D41, and 5Y9T). The selected hits were docked to the mutant proteins and the binding was analyzed by aligning them on the co-crystal ligands of the respective proteins. The alignment of the docked hits with the mutant proteins revealed that the hits occupied the same space in the binding site of the protein as co-crystal ligand (Fig 6). Thus, the plausible binding modes of the selected compounds were subjected towards the stability analysis by employing the MD Simulation study.

### MD simulation

#### RMSD

**Fig 7:**
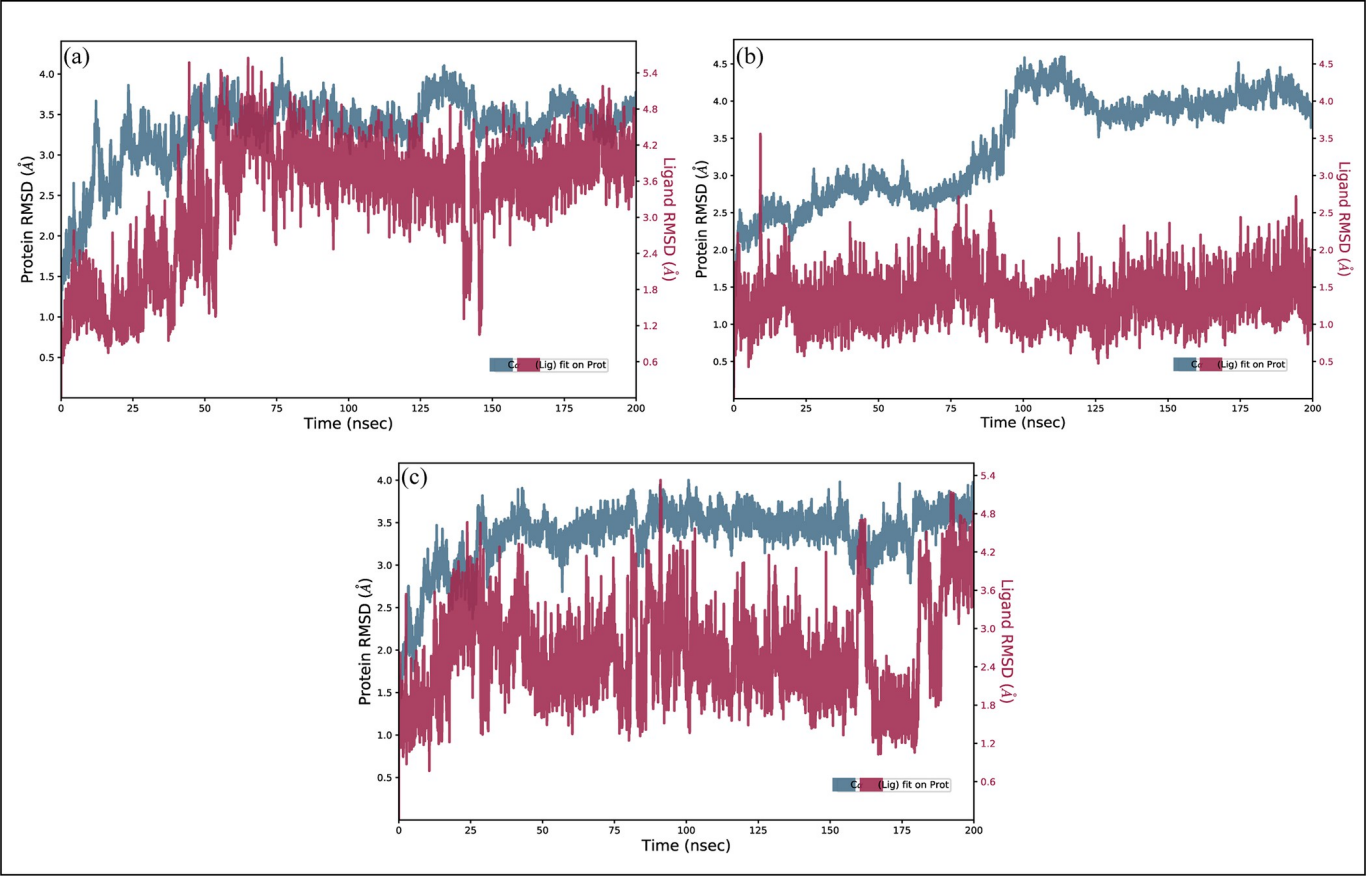
The RMSD of EGFR complexes calculated during 200 ns simulation. (a) MCULE-6473175764, (b) CSC048452634, (c) CSC070083626.

**Fig 8:**
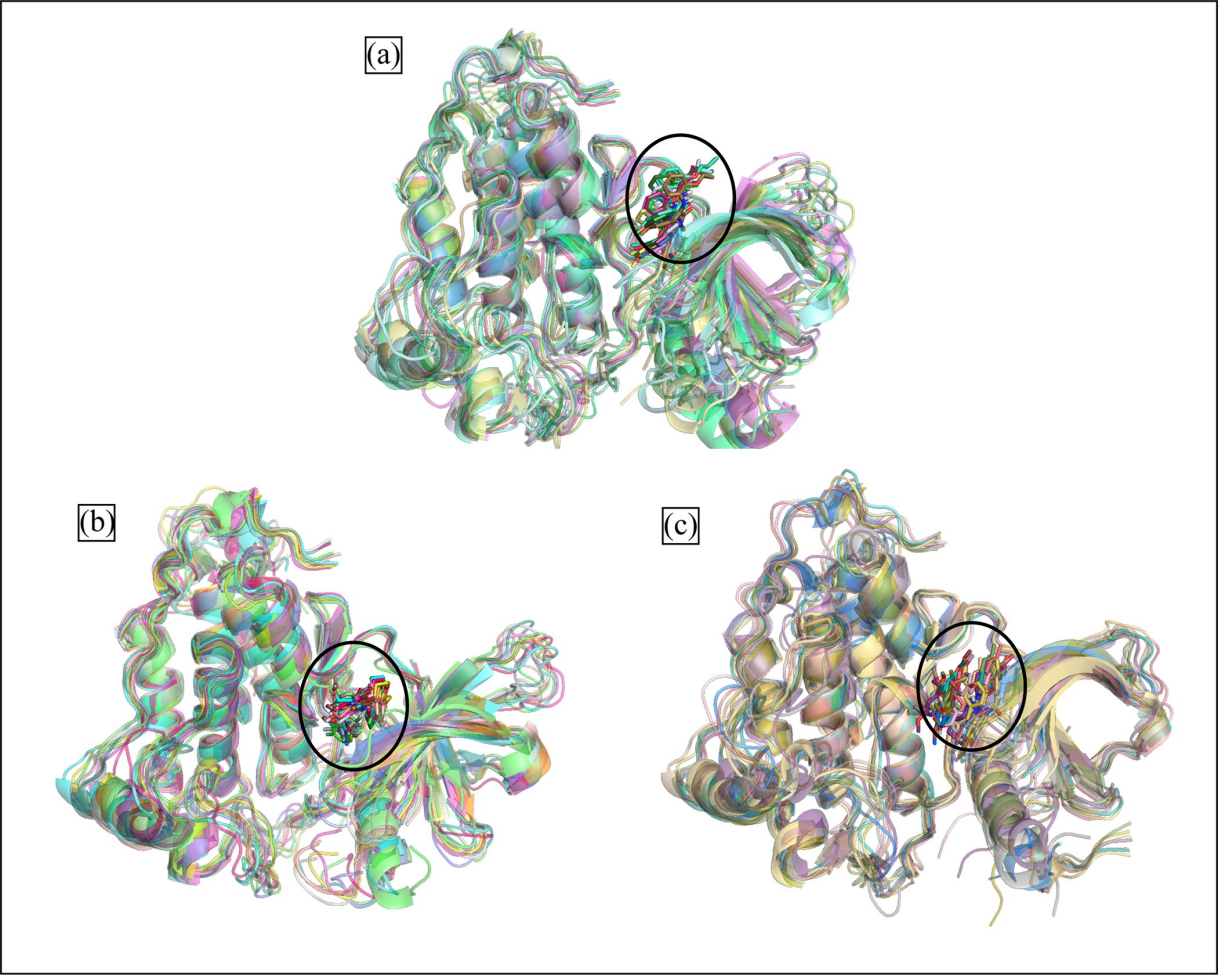
The aligned snapshots of EGFR complexes extracted during 200 ns simulation. (a) MCULE-6473175764, (b) CSC048452634, (c) CSC070083626.

To confirm the stability of the protein-ligand complexes, Molecular Dynamics (MD) simulation of 200ns was employed to investigate the binding sites of those selected compounds against the EGFR receptor. The RMSD of the carbon alpha (C) atoms was calculated in order to look into the complexes’ deviations and general structural changes during the simulation [30]. The **MCULE-6473175764** complex’s RMSD readings gradually increased to 4 Å at 50 ns and stayed in the 3.5–4 Å range until 150 ns. The RMSD readings dropped to 3 Å after 150 ns and stayed that way until the simulation was over. The RMSD of ligand was perfectly aligned on protein during simulation (Fig 7A). The RMSD of **CSC048452634** remained in the range of 3 Å in the first half of simulation and then increased to 4.5 Å at 100 ns but it again decreased to 4 Å at 125 ns. The RMSD stabilized in the 4 Å range after 125 ns. Compared to the protein, the ligand fit’s RMSD was less (Fig 7B). Lastly, the RMSD of **CSC070083626** attained stability in the range of 3.5 at 25 ns and stayed there until the simulation’s end, while the RMSD of ligand fit was slightly lower than the protein RMSD (Fig 7C). Furthermore, the snapshots of MD trajectories were extracted at 0, 20, 40, 60, 80, 100, 120, 140, 160, 180, and 200 ns and aligned to analyze the position of the ligands during the simulation. It was observed that the compounds **MCULE-6473175764, CSC048452634,** and **CSC070083626** remained tightly bound to the protein during simulation (Fig 8).

#### RMSF

**Fig 9:**
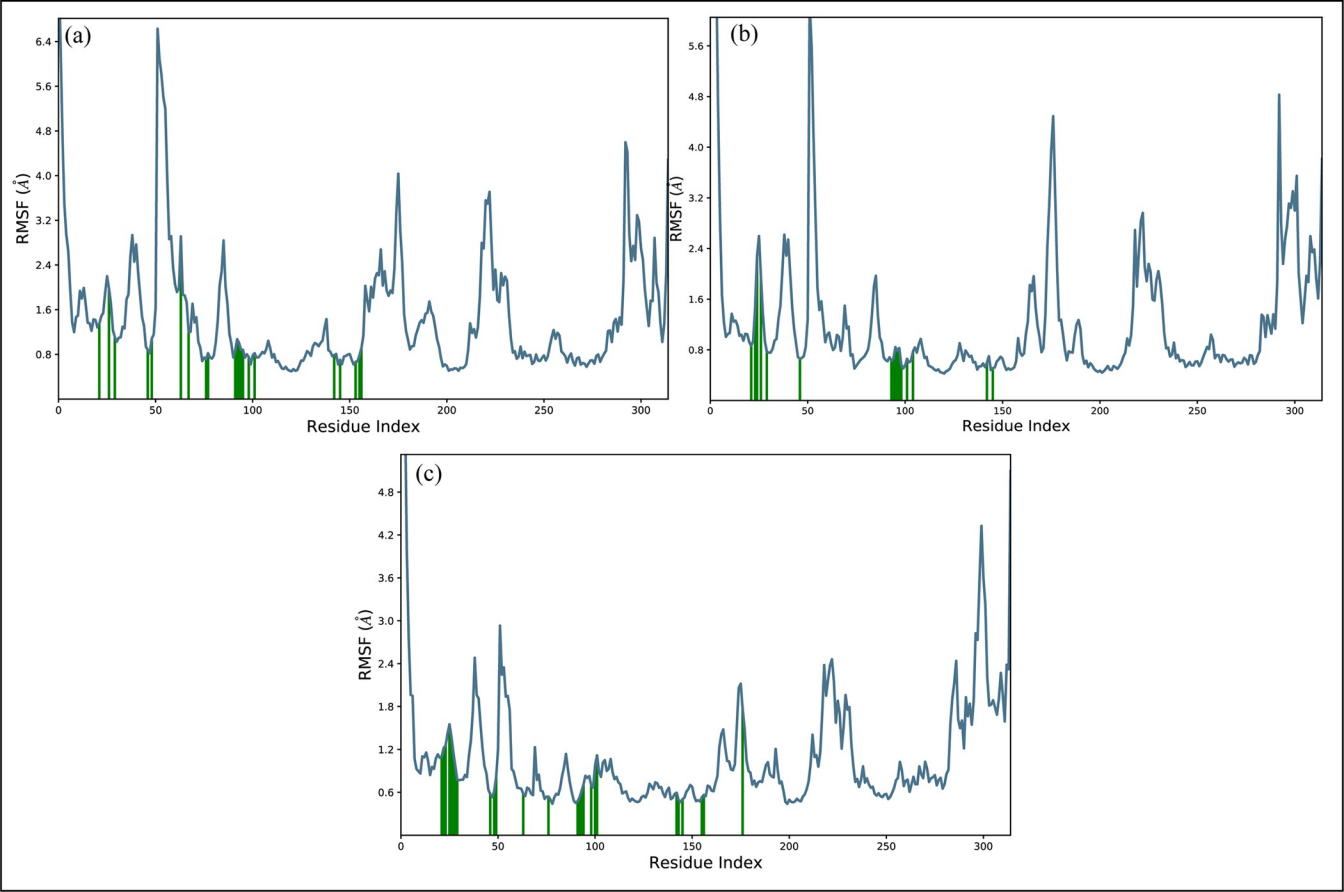
The residual fluctuations of the EGFR receptor upon binding of the selected compounds. (a) MCULE-6473175764, (b) CSC048452634, (c) CSC070083626.

**Fig 10:**
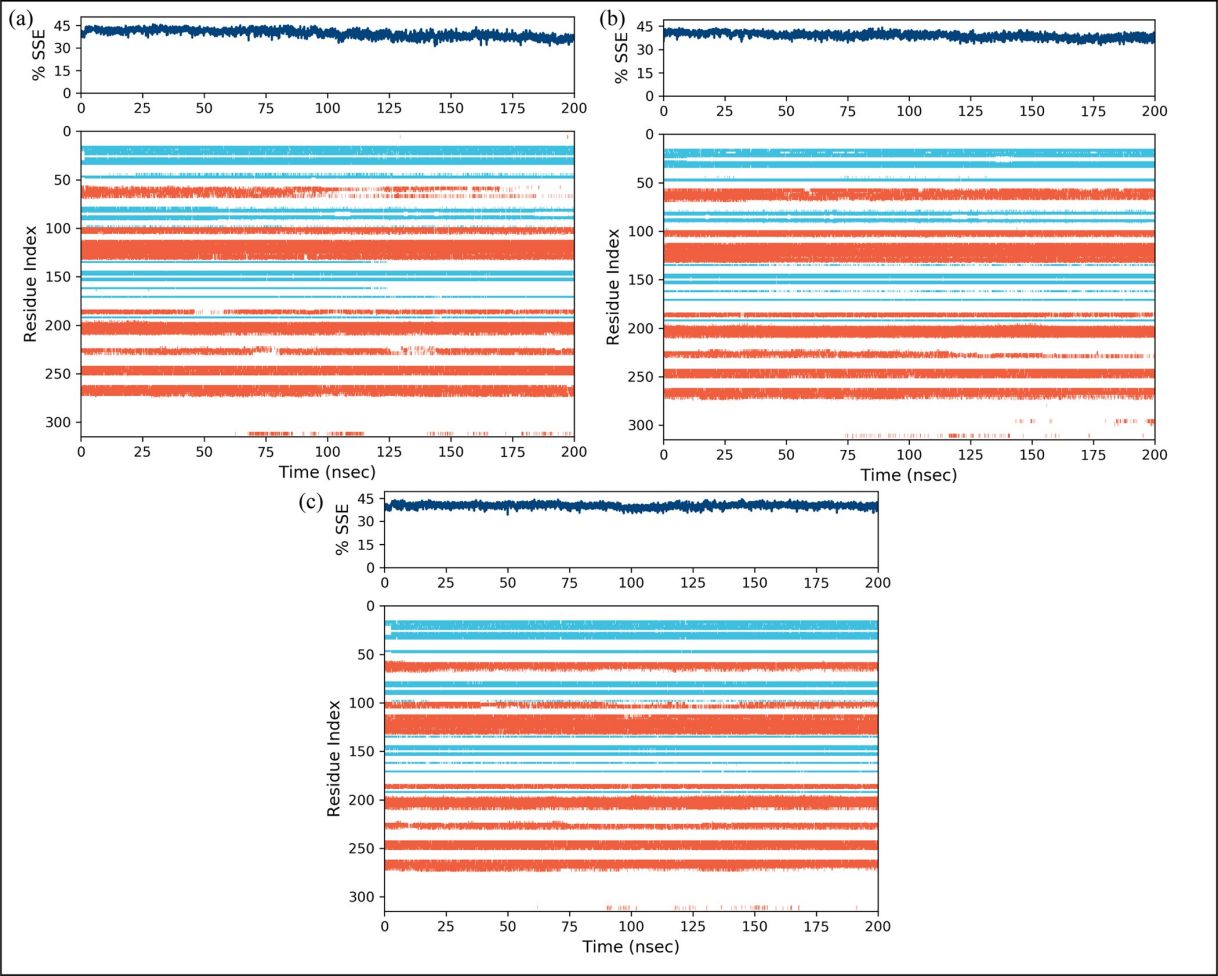
The percentage of secondary structure elements of EGFR receptor upon binding of the selected compounds. (a) MCULE-6473175764, (b) CSC048452634, (c) CSC070083626.

Root mean square fluctuations (RMSF) values have been calculated in order to identify the fluctuation of the proteins while they are bound to the ligands [31]. For each protein residue over the simulation period, RMSF values give detailed information on the residue’s mobility and flexibility. Based on the expected RMSF values, most protein residues changed very slightly during the simulation, which was less than 2Å. This suggests that these residues maintained their relative stability and stiffness while the ligands were present. The RMSF values of the protein’s loop regions, which include residues that go from 50 to 60, 170 to 185, 220 to 235, and 290 to 310, were higher and reached around 6Å (Fig 9). The creation of a stable complex was shown by the green lines that depicted the interactions between the ligand and protein residues. The RMSF values of the loop parts were greater, indicating that these areas noticed more significant fluctuations and may have had dynamic interactions with the ligands. Most protein residues showed slight changes, but loop parts showed larger degrees of flexibility. Overall, the RMSF values are compatible with the idea of a stable protein-ligand complex. Furthermore, the secondary structures elements were estimated (Fig 10). The blue regions showed the presence of alpha helices while orange color indicates the beta sheets. The loops were exhibited in white color. During the simulation, it was estimated that the secondary structures did not show fluctuations and remained stable upon binding of the ligands.

### Protein-ligand contacts

**Fig 11:**
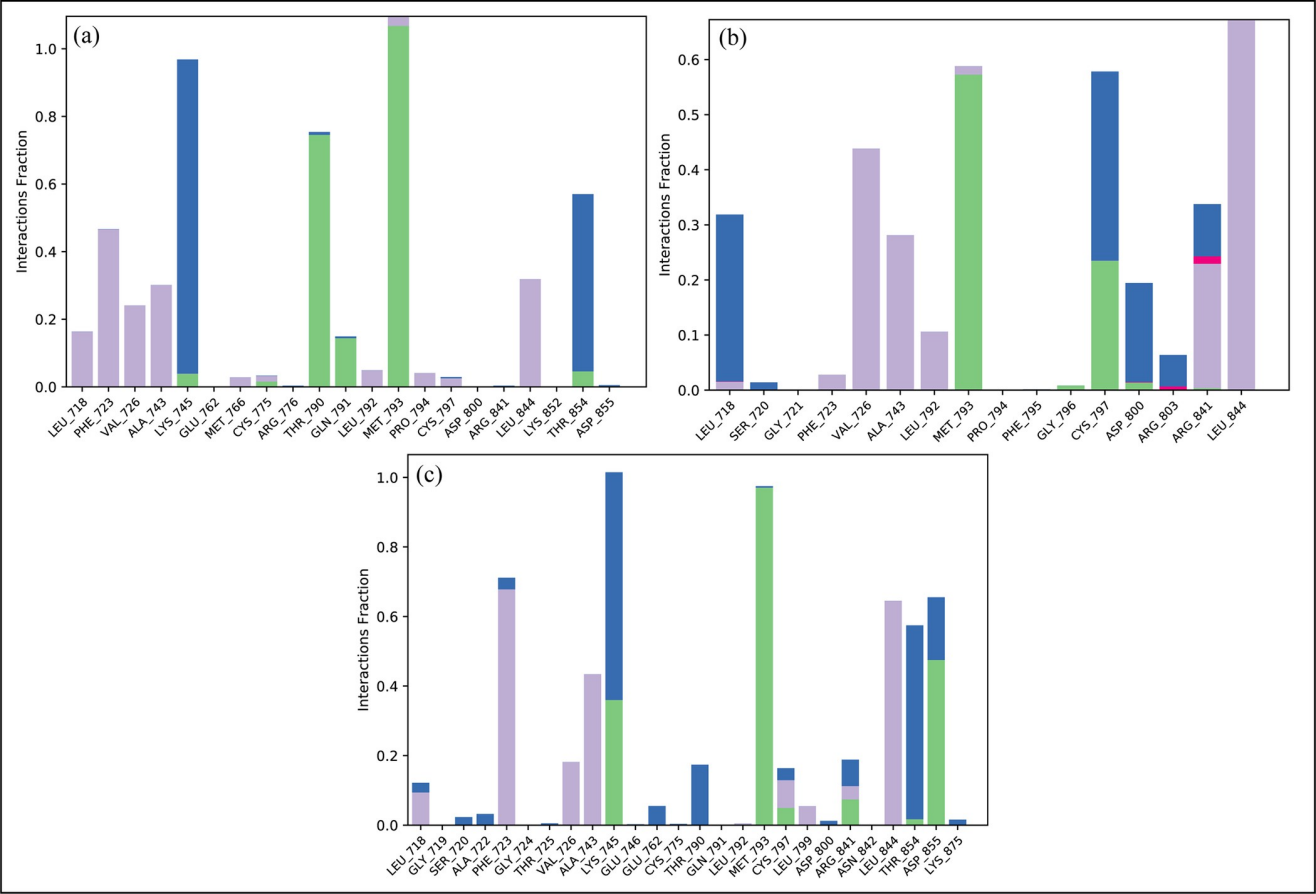
The interaction of protein-ligand during MDS. (a) MCULE-6473175764, (b) CSC048452634, (c) CSC070083626. The residues that interact are shown as tall, stacked bars. Hydrogen bonding is represented by green bars, hydrophobic interactions by grey bars, and water bridges by blue bars.

The MD Simulation analysis showed that ionic, hydrogen, and hydrophobic bonds were the most important types of interactions between the ligands and the protein. The functional properties of the protein-ligand complex are stabilized and regulated by these interactions. Residues that form hydrogen bonds with **MCULE-6473175764** were Lys745, Thr790, Met793, and Thr854 (Fig 11A). In the **CSC048452634** complex, the residues involved in hydrogen bonding were Met793, Gly796, cys797, and Asp800 (Fig 11B). In the **CSC070083626** complex, the hydrogen bonding interactions involved Lys745, Met793, Cys797, Arg841, Thr854, and Asp855 (Fig 11C). These hydrogen bonding interactions, which were displayed during the MD simulations, not only highlighted the specific residues that were crucial for stabilizing the protein-ligand complexes, but they also provided insight into the crucial interactions that maintain the complexes’ general stability and binding affinity.

### Hydrogen bonding and distance measurement

**Fig 12:**
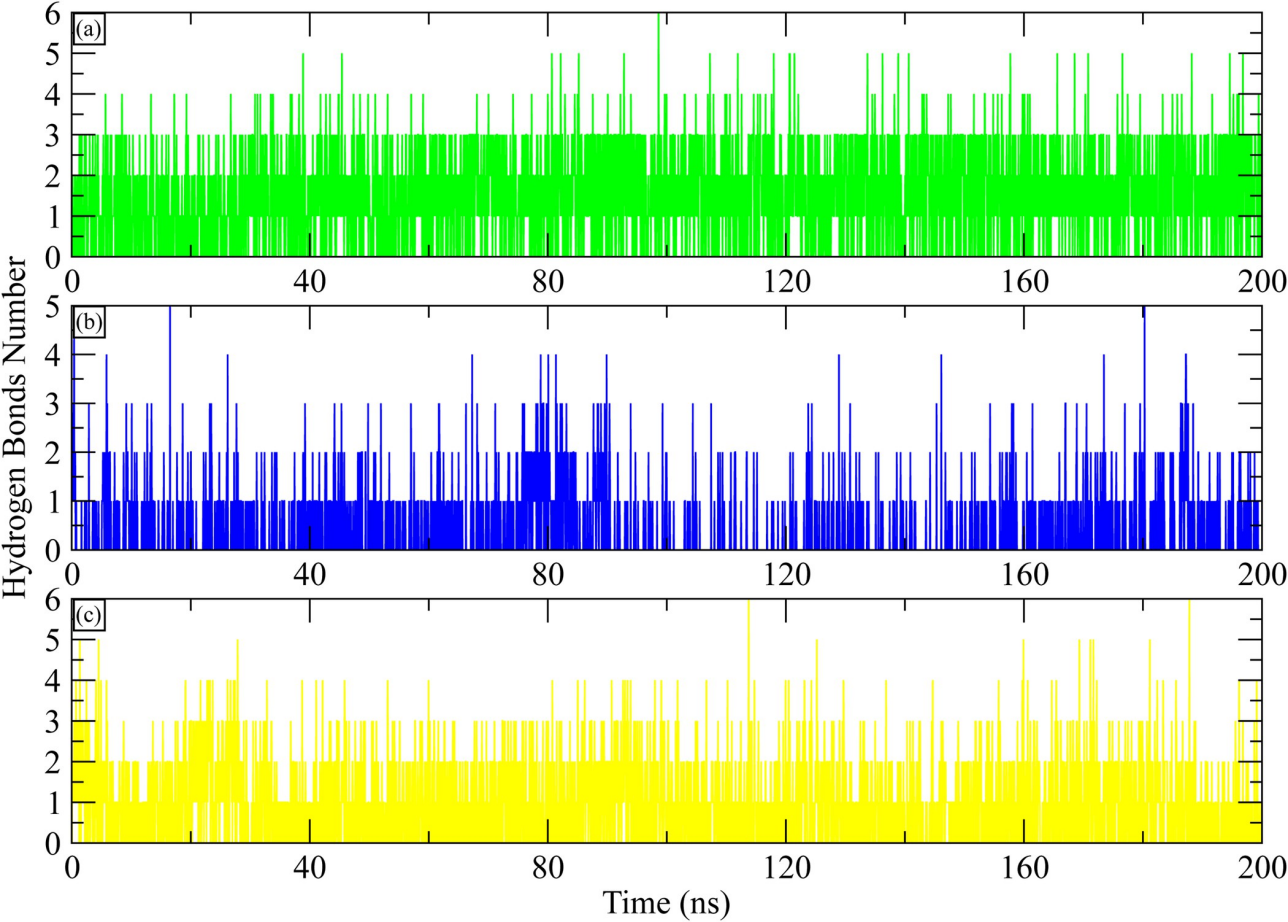
The number of hydrogen bonds between EGFR and selected ligand calculated during 200 ns simulation. (a) MCULE-6473175764, (b) CSC048452634, (c) CSC070083626.

**Fig 13:**
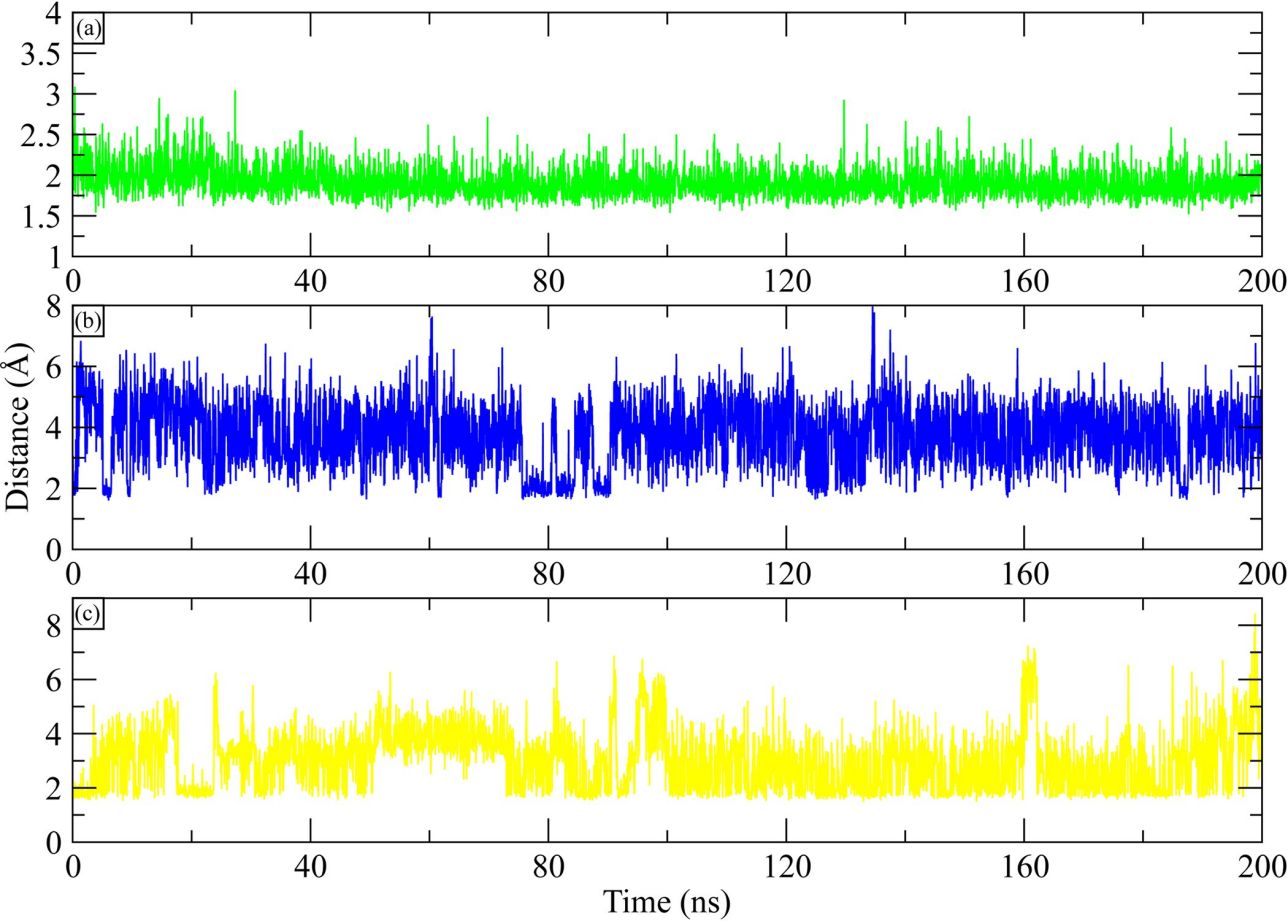
The distance between the key residues of EGFR complexes during 200 ns simulation. (a) MCULE-6473175764, (b) CSC048452634, (c) CSC070083626.

A key factor in the stability of the protein-ligand complex is hydrogen bonding. As a result, throughout the simulation, the number of hydrogen bonds between the ligand and the active site residues was determined. The hydrogen bonding plots indicate that **MCULE-6473175764** made at least three hydrogen bonds throughout the simulation. Some frames showed five hydrogen bonds while six bonds were also observed (Fig 12A). **CSC048452634** at least made two hydrogen bonds throughout the simulation. (Fig 12B). While **CSC070083626** made at least three hydrogen bonds during simulation (Fig 12C). Further, the distance between hydrogen bond forming residues and ligand atoms was calculated during the simulation. The initial distance between hydroxyl group of **MCULE-6473175764** and HG1 hydrogen of Thr854 was 1.8 Å and it remained in the range of 2–2.5 Å throughout the simulation (Fig 13A). Similarly, the initial distance between hydroxyl group of **CSC048452634** and OD2 oxygen of Asp800 was 1.84 Å which increased to 6 at 5 ns, throughout the simulation, it remained in the range of 4–6 Å (Fig 13B). Lastly, the distance between the hydroxyl group of **CSC070083626** and OD2 oxygen of Asp855 was 1.73 Å at the start, which increased to 4 Å and remained in this range in first half of simulation, while in second half the distance increased to 6 Å (Fig 13C).

### MMGBSA

Molecular mechanics Generalized Born surface area (MM/GBSA) method was used to calculate the total binding free energy (ΔG_total_). ΔG_total_ value is usually used to estimate the stability of protein-ligand complex [32]. It was computed as a sum of protein-ligand complex and the difference of protein and its ligands free energies. The total binding free energy estimated using MM/GBSA model is the outcome of the contribution of various protein-ligand interactions such as van der Waals energy (**ΔE**_**vdW**_), electrostatic energy (**ΔE**_**ele**_), **ΔG**_**GB**_ (electrostatic contribution to solvation free energy by Generalized Born). The ΔE_vdW_ contribution of MCULE-6473175764 complex was more than remaining two complexes which was -62.46 kcal/mol while the electrostatic energy of CSC048452634 complex was more. The Generalized Born solvation energy of CSC048452634 complex was also greater than other complexes. The contribution of other energy components is shown in Table 6.

**Table 6:** The MM/GBSA calculations of the selected complexes.

Energy components	MCULE-6473175764	CSC048452634	CSC070083626
**ΔE**_**vdW**_	-62.46 ± 0.22	-49.02 ± 0.19	-58.77 ± 0.24
**ΔE**_**ele**_	-4.78 ± 0.19	-14.18 ± 0.29	-7.48 ± 0.22
**ΔE**_**GB**_	26.23 ± 0.18	32.42 ± 0.29	24.14 ± 0.19
**ΔE**_**surf**_	-6.91 ± 0.01	-5.38 ± 0.01	-6.57 ± 0.01
**ΔG**_**gas**_	-67.24 ± 0.30	-63.21 ± 0.32	-66.25 ± 0.33
**ΔG**_**solv**_	19.32 ± 0.18	27.04 ± 0.28	17.56 ± 0.19
**ΔG**_**total**_	-47.92 ± 0.28	-36.16 ± 0.19	-48.69 ± 0.30

## Discussion

Despite the initial success of EGFR inhibitors, resistance frequently develops over time, limiting their long-term effectiveness. Cancer cells can adapt through a variety of mechanisms, including secondary mutations in the EGFR gene and alternative signaling pathways. To overcome or delay the development of resistance, novel inhibitors with different mechanisms of action are required [16,33]. The combination of pharmacophore-based virtual screening, molecular docking, ADMET analysis, and MD simulation provides a thorough and systematic approach to drug discovery. This study focuses on EGFR and seeks to identify novel compounds with strong therapeutic potential.

The nine databases were first virtually screened using pharmacophores, and the screened hits were then docked to the EGFR active site to determine the best binding modes. Pharmacophore-based virtual screening identifies chemical features required for molecular recognition and binding. Pharmacophore models are created from commercial databases to filter compounds based on their ability to match critical structural features required for EGFR inhibition. This step narrows the pool of compounds, ensuring that only those containing the desired pharmacophoric elements are considered for further investigation [34]. A ligand-based pharmacophore model was developed of Epidermal growth factor receptor. A ligand-based virtual screening of the nine databases was performed based on these characteristics. This process involved evaluating each database to find compounds that matched a predefined pharmacophore model, which represents the essential features required for effective binding to the target receptor [35]. From this extensive screening, 1271 compounds (hits) were identified that met the stringent criteria set by the pharmacophore model.

EGFR protein crystal structure was obtained from the PDB database (PDB ID: 7AEI) and prepared. The hit compounds identified during virtual screening were docked to the prepared EGFR receptor to predict binding affinities using the glide tool’s standard precision mode. This step aids in predicting the compounds’ potential efficacy in inhibiting EGFR enzymatic activity [36]. The glide tool assigns a binding affinity score to each molecule based on its evaluation of the binding interactions with the EGFR active site. Binding affinity is measured in kcal/mol, with negative values indicating stronger binding interactions and better ligand-receptor complex stability. Based on the docking results, the top ten compounds with the highest binding affinities were chosen for further study. These chemicals’ binding affinities ranged from -7.691 to -7.338 kcal/mol, better than the binding affinities of the co-crystal ligand and know ATP-competitive EGFR inhibitors (Gefitinib, Erlotinib, Afatinib, Osimertinib [37,38]) indicating that they generated extremely stable connections with the EGFR receptor. This range of binding affinities shows that these chemicals are excellent candidates for inhibiting EGFR, as they are likely to occupy the active site and disrupt the receptor’s enzymatic function.

The molecular interactions of the selected hit compounds with the EGFR receptor binding pocket were thoroughly investigated to understand their high binding affinities and docking scores. The study found that a variety of interactions were important in the stabilization of ligand-receptor complexes. Conventional hydrogen bonds, in which hydrogen atoms form strong, directional bonds with electronegative atoms such as oxygen or nitrogen, were most prevalent and significantly contributed to binding specificity and strength [39]. Though weaker, carbon hydrogen bonds helped to maintain stability by forming between electronegative atoms in the receptor and hydrogen atoms connected to carbons. Extra non-covalent stability was supplied by van der Waals interactions, which were the outcome of atoms being near to one another. Pi-Sulfur interactions occur when ligands’ aromatic rings contact with sulfur atoms, improving binding due to sulfur’s special electronic properties. A combination of aromatic stacking and electron cloud interactions between aromatic rings stabilized the complexes further through Pi-Pi Stacked and Pi-Sigma interactions [40]. The last factor that affected the overall binding affinity was the alkyl interaction that developed between the receptor’s hydrophobic areas and the ligands’ non-polar alkyl groups. These many interactions together generated the top candidate compounds’ high docking scores and binding affinities, highlighting their potential as powerful inhibitors of EGFR.

Additionally, evaluating ADMET properties is an important aspect of drug development [41]. The ADMET (Absorption, Distribution, Metabolism, Excretion, and Toxicity) characteristics of the chosen compounds were assessed to ensure that they had good pharmacokinetic and safety profiles, which are required for therapeutic development [42]. The investigation revealed that all the compounds had ADMET values within acceptable limits, implying that they had high potential as drug candidates. Three of these compounds—MCULE-6473175764, CSC048452634, and CSC070083626—had extremely high QPPCaco values. QPPCaco is a predictive indicator for intestinal permeability, which is a key determinant in oral bioavailability. Compounds having higher QPPCaco values are more likely to be absorbed through the intestinal lining, which increases their potential as orally given medicines. These three compounds were chosen for additional stability testing due to their high QPPCaco values.

The dynamic behavior of EGFR-inhibitor complexes over time is also investigated using Molecular Dynamics (MD) simulations. Researchers can use this computational technique to investigate the stability and flexibility of binding interactions, which provides valuable information on structural changes that may affect the inhibitor’s efficacy [43]. Based on molecular dynamics simulations, these substances persisted as potent inhibitors inside the protein binding pocket. All these findings suggest that the selected hit compounds could work as lead compounds and inhibit EGFR’s biological activity. This multifaceted technique not only improves the precision and efficiency of drug discovery procedures, but also paves the way for future research on other essential biological receptors. Future research may broaden this integrative strategy to include more targets, overcoming present study limitations by experimental confirmation and the use of newer databases, accelerating the development of new therapies for EGFR-related illnesses.

## Conclusions

This study focuses on the EGFR, seeks to identify novel compounds with strong therapeutic potential. The use of commercial databases broadens the scope and diversity of compounds considered, increasing the likelihood of finding potent and selective EGFR inhibitors. Looking ahead, future research could expand this methodology to other critical biological targets, enhancing its applicability across various diseases. Despite its success, the study faces limitations, including computational intensity, reliance on predictive accuracy without experimental validation, and potential gaps in commercial databases. To address these, leveraging high-performance computing, collaborating with experimental labs for validation, and incorporating additional novel databases could enhance the robustness and effectiveness of this comprehensive drug discovery approach.

## References

[1] Seshacharyulu P., et al., Targeting the EGFR signaling pathway in cancer therapy. Expert opinion on therapeutic targets, 2012. 16(1): p. 15–31. doi: 10.1517/14728222.2011.648617 22239438 PMC3291787

[2] Harari P., Epidermal growth factor receptor inhibition strategies in oncology. Endocrine-related cancer, 2004. 11(4): p. 689–708. doi: 10.1677/erc.1.00600 15613446

[3] Bhat S.S. and Prasad S.K., In silico Screening of Violacein as an epidermal growth factor receptor inhibitor. International Journal of Health and Allied Sciences, 2022. 11(1): p. 6.

[4] Yewale C., et al., Epidermal growth factor receptor targeting in cancer: a review of trends and strategies. Biomaterials, 2013. 34(34): p. 8690–8707. doi: 10.1016/j.biomaterials.2013.07.100 23953842

[5] Passaro A., et al., Recent advances on the role of EGFR tyrosine kinase inhibitors in the management of NSCLC with uncommon, non exon 20 insertions, EGFR mutations. Journal of Thoracic Oncology, 2021. 16(5): p. 764–773. doi: 10.1016/j.jtho.2020.12.002 33333327

[6] Sequist L.V., et al., Osimertinib plus savolitinib in patients with EGFR mutation-positive, MET-amplified, non-small-cell lung cancer after progression on EGFR tyrosine kinase inhibitors: interim results from a multicentre, open-label, phase 1b study. The Lancet Oncology, 2020. 21(3): p. 373–386. doi: 10.1016/S1470-2045(19)30785-5 32027846

[7] Park S.-Y., Kim Y.M., and Pyo H., Gefitinib radiosensitizes non-small cell lung cancer cells through inhibition of ataxia telangiectasia mutated. Molecular cancer, 2010. 9(1): p. 1–12. doi: 10.1186/1476-4598-9-222 20731837 PMC2936341

[8] Anderson N.G., et al., ZD1839 (Iressa), a novel epidermal growth factor receptor (EGFR) tyrosine kinase inhibitor, potently inhibits the growth of EGFR‐positive cancer cell lines with or without erbB2 overexpression. International journal of cancer, 2001. 94(6): p. 774–782. doi: 10.1002/ijc.1557 11745477

[9] Lin N.U., et al., A phase II study of afatinib (BIBW 2992), an irreversible ErbB family blocker, in patients with HER2-positive metastatic breast cancer progressing after trastuzumab. Breast cancer research and treatment, 2012. 133: p. 1057–1065. doi: 10.1007/s10549-012-2003-y 22418700 PMC3387495

[10] Lazzari C., et al., Mechanisms of resistance to osimertinib. Journal of Thoracic Disease, 2020. 12(5): p. 2851. doi: 10.21037/jtd.2019.08.30 32642198 PMC7330330

[11] Mok T.S., et al., Osimertinib or platinum–pemetrexed in EGFR T790M–positive lung cancer. New England Journal of Medicine, 2017. 376(7): p. 629–640. doi: 10.1056/NEJMoa1612674 27959700 PMC6762027

[12] Yin B., et al., Natural products as important tyrosine kinase inhibitors. European journal of medicinal chemistry, 2019. 182: p. 111664. doi: 10.1016/j.ejmech.2019.111664 31494475

[13] Abdelgawad M.A., et al., Novel phenolic compounds as potential dual EGFR and COX-2 inhibitors: Design, semisynthesis, in vitro biological evaluation and in silico Insights. Drug design, development and therapy, 2021: p. 2325–2337. doi: 10.2147/DDDT.S310820 34103896 PMC8178614

[14] Abou-Zied H.A., et al., EGFR inhibitors and apoptotic inducers: Design, synthesis, anticancer activity and docking studies of novel xanthine derivatives carrying chalcone moiety as hybrid molecules. Bioorganic chemistry, 2019. 89: p. 102997. doi: 10.1016/j.bioorg.2019.102997 31136902

[15] Acevedo C.H., Scotti L., and Scotti M.T., In silico studies designed to select sesquiterpene lactones with potential antichagasic activity from an in‐house asteraceae database. ChemMedChem, 2018. 13(6): p. 634–645. doi: 10.1002/cmdc.201700743 29323468

[16] Wheeler D.L., Dunn E.F., and Harari P.M., Understanding resistance to EGFR inhibitors—impact on future treatment strategies. Nature reviews Clinical oncology, 2010. 7(9): p. 493–507. doi: 10.1038/nrclinonc.2010.97 20551942 PMC2929287

[17] Naqvi A.A., et al., Advancements in docking and molecular dynamics simulations towards ligand-receptor interactions and structure-function relationships. 2018. 18(20): p. 1755–1768. doi: 10.2174/1568026618666181025114157 30360721

[18] Sunseri J. and Koes D.R.J.N.a.r, Pharmit: interactive exploration of chemical space. 2016. 44(W1): p. W442–W448. doi: 10.1093/nar/gkw287 27095195 PMC4987880

[19] Prabitha P., et al., Multi-conformational frame from molecular dynamics as a structure-based pharmacophore model for mapping, screening and identifying ligands against PPAR-γ: a new protocol to develop promising candidates. 2022. 40(6): p. 2663–2673. 10.1080/07391102.2020.1841677 33140698

[20] Oduselu G.O., Ajani O.O., and Ajamma Y.U., Structure-Based drug design in discovering target specific drugs against plasmodium falciparum adenylosuccinate lyase. 2021.

[21] LigPrep, LigPrep. 2018, Schrödinger, LLC.

[22] Shivakumar D., et al., Improving the prediction of absolute solvation free energies using the next generation OPLS force field. 2012. 8(8): p. 2553–2558. doi: 10.1021/ct300203w 26592101

[23] Schrödinger L.J.S.S., Schrödinger, LLC; New York, NY: 2017. 2017. 2: p. 2017–1.

[24] Kim M.O., et al., Effects of histidine protonation and rotameric states on virtual screening of M. tuberculosis RmlC. 2013. 27(3): p. 235–246. 10.1007/s10822-013-9643-9 PMC3639364 23579613

[25] Friesner R.A., et al., Glide: a new approach for rapid, accurate docking and scoring. 1. Method and assessment of docking accuracy. 2004. 47(7): p. 1739–1749. 10.1021/jm0306430 15027865

[26] Mali S.N. and Chaudhari H.K.J.O.P.S.J., Computational studies on imidazo [1, 2-a] pyridine-3-carboxamide analogues as antimycobacterial agents: Common pharmacophore generation, atom-based 3D-QSAR, molecular dynamics simulation, QikProp, molecular docking and prime MMGBSA approaches. 2018. 5(1).

[27] Bowers K.J., et al. Scalable algorithms for molecular dynamics simulations on commodity clusters. in Proceedings of the 2006 ACM/IEEE Conference on Supercomputing. 2006.

[28] Price D.J. and Brooks C.L.J.T.J.o.c.p III, A modified TIP3P water potential for simulation with Ewald summation. 2004. 121(20): p. 10096–10103. doi: 10.1063/1.1808117 15549884

[29] Thillainayagam M., et al., In-Silico molecular docking and simulation studies on novel chalcone and flavone hybrid derivatives with 1, 2, 3-triazole linkage as vital inhibitors of Plasmodium falciparum dihydroorotate dehydrogenase. 2018. 36(15): p. 3993–4009. doi: 10.1080/07391102.2017.1404935 29132266

[30] Sargsyan K., et al., How molecular size impacts RMSD applications in molecular dynamics simulations. 2017. 13(4): p. 1518–1524. doi: 10.1021/acs.jctc.7b00028 28267328

[31] Martínez L.J.P.o., Automatic identification of mobile and rigid substructures in molecular dynamics simulations and fractional structural fluctuation analysis. 2015. 10(3): p. e0119264. doi: 10.1371/journal.pone.0119264 25816325 PMC4376797

[32] Du J., et al., Molecular modeling study of checkpoint kinase 1 inhibitors by multiple docking strategies and prime/MM–GBSA calculation. 2011. 32(13): p. 2800–2809. doi: 10.1002/jcc.21859 21717478

[33] Lurje G. and Lenz H.-J., EGFR signaling and drug discovery. Oncology, 2010. 77(6): p. 400–410. 10.1159/000279388 20130423

[34] Muhammed M.T. and Esin A.-Y., Pharmacophore modeling in drug discovery: methodology and current status. Journal of the Turkish Chemical Society Section A: Chemistry, 2021. 8(3): p. 749–762.

[35] Banegas-Luna A.-J., Cerón-Carrasco J.P., and Pérez-Sánchez H., A review of ligand-based virtual screening web tools and screening algorithms in large molecular databases in the age of big data. Future medicinal chemistry, 2018. 10(22): p. 2641–2658. doi: 10.4155/fmc-2018-0076 30499744

[36] Jakhar R., et al., Relevance of molecular docking studies in drug designing. Current Bioinformatics, 2020. 15(4): p. 270–278.

[37] Spellmon N., Li C., and Yang Z.J.J.o.t.d, Allosterically targeting EGFR drug-resistance gatekeeper mutations. 2017. 9(7): p. 1756. doi: 10.21037/jtd.2017.06.43 28839955 PMC5542946

[38] Beyett T.S., et al., Molecular basis for cooperative binding and synergy of ATP-site and allosteric EGFR inhibitors. 2022. 13(1): p. 2530. doi: 10.1038/s41467-022-30258-y 35534503 PMC9085736

[39] Alkorta I., Elguero J., and Frontera A., Not only hydrogen bonds: Other noncovalent interactions. Crystals, 2020. 10(3): p. 180.

[40] Mitra D., et al., C-halogen… pi interactions in nucleic acids: A database study. Journal of Chemical Sciences, 2020. 132: p. 1–6.

[41] Jia C.-Y., et al., A drug-likeness toolbox facilitates ADMET study in drug discovery. Drug discovery today, 2020. 25(1): p. 248–258. doi: 10.1016/j.drudis.2019.10.014 31705979

[42] Dulsat J., et al., Evaluation of free online ADMET tools for academic or small biotech environments. Molecules, 2023. 28(2): p. 776. doi: 10.3390/molecules28020776 36677832 PMC9864198

[43] Salo-Ahen O.M., et al., Molecular dynamics simulations in drug discovery and pharmaceutical development. Processes, 2020. 9(1): p. 71.

